# Low Dietary Magnesium and Overweight/Obesity in a Mediterranean Population: A Detrimental Synergy for the Development of Hypertension. The SUN Project

**DOI:** 10.3390/nu13010125

**Published:** 2020-12-31

**Authors:** Ligia J. Dominguez, Alfredo Gea, Liz Ruiz-Estigarribia, Carmen Sayón-Orea, Ujue Fresán, Mario Barbagallo, Miguel Ruiz-Canela, Miguel A. Martínez-González

**Affiliations:** 1 Geriatric Unit, Department of Internal Medicine and Geriatrics, University of Palermo, 90127 Palermo, Italy; mario.barbagallo@unipa.it; 2 Department of Preventive Medicine and Public Health, University of Navarra-IDISNA, 31008 Pamplona, Spain; ageas@unav.es (A.G.); lruiz.29@alumni.unav.es (L.R.-E.); msayon@unav.es (C.S.-O.); mcanela@unav.es (M.R.-C.); mamartinez@unav.es (M.A.M.-G.); 3 CIBER Fisiopatología de la Obesidad y Nutrición (CIBERobn), Instituto de Salud Carlos III, 28029 Madrid, Spain; 4 Public Health Institute, 31003 Navarra, Spain; 5 eHealth Group, ISGlobal, 08036 Barcelona, Spain; ujuefresan@gmail.com; 6 Department of Nutrition, Harvard TH Chan School of Public Health, Boston, MA 02115, USA

**Keywords:** magnesium, hypertension, obesity, overweight, diet, cohort studies

## Abstract

Hypertension is the strongest independent modifiable risk factor for cardiovascular disease. We aimed to investigate the association of magnesium intake with incident hypertension in a Mediterranean population, and the potential modification of this association by body mass index (BMI). We assessed 14,057 participants of the SUN (Seguimiento Universidad de Navarra) prospective cohort (67.0% women) initially free of hypertension. At baseline, a validated 136-item food frequency questionnaire was administered. We used Cox models adjusted for multiple socio-demographic, anthropometric, and lifestyle factors, and prevalent conditions present at baseline. Among a mean 9.6 years of follow-up we observed 1406 incident cases of medically diagnosed hypertension. An inverse association in multivariable-adjusted models was observed for progressively higher magnesium intake up to 500 mg/d vs. intake < 200 mg/d, which was greater among those with a BMI > 27 kg/m^2^. Lean participants with magnesium intake < 200 mg/d vs. >200 mg/d also had a higher risk of incident hypertension. Adherence to the Mediterranean diet did not modify these associations. In conclusion, dietary magnesium intake < 200 mg/d was independently associated with a higher risk of developing hypertension in a Mediterranean cohort, stronger for overweight/obese participants. Our results emphasize the importance of encouraging the consumption of magnesium-rich foods (vegetables, nuts, whole cereals, legumes) in order to prevent hypertension.

## 1. Introduction

Hypertension is the strongest independent and modifiable risk factor for heart failure, stroke, myocardial infarction, chronic kidney disease, and cognitive decline [1]. In 2015, hypertension was associated with 4.9, 2.0, and 1.5 million deaths due to ischemic heart disease and hemorrhagic and ischemic stroke, respectively [2]. Currently, 1.13 billion adults have hypertension worldwide according to the World Health Organization (WHO) [3] and its prevalence is still increasing [4]. Therefore, public health preventive strategies are urgently needed to fight the hypertension pandemic.

Magnesium is involved in fundamental cellular processes comprising adenosine triphophosphate-dependent biochemical reactions, deoxyribonucleic acid synthesis, ribonucleic acid expression, muscular and neural cell signaling, glucose metabolism, and blood pressure control [5,6]. Magnesium is involved in the regulation of blood pressure by diverse mechanisms including vascular tone and reactivity modulation acting as a calcium antagonist [7,8], the renin–angiotensin–aldosterone system [9], endothelial function [10,11,12], vascular remodeling and stiffness [13], and catecholamine release [14].

Magnesium is abundant in nuts, in green leafy vegetables, in legumes, and in whole cereals, whereas it is practically absent in processed foods [15]. Dietary magnesium intake is insufficient in a large proportion of European and US populations where Western dietary patterns full of processed foods are usual [16,17,18,19]. The Dietary Guidelines for Americans recommend a daily intake of 420 mg of magnesium for men and 320 mg for women [20], but estimates indicate that over 60% of Americans are below this recommendation [18].

Magnesium has to be consumed regularly to prevent deficiency, which is associated with oxidative stress and low-grade inflammation [12,21,22], and with an increased risk of a number of clinical conditions including hypertension and stroke [5,7,23]. Previous studies have shown inverse associations of magnesium intake in the diet with the risk of hypertension [24,25,26,27,28,29,30,31], with fewer reporting negative or inconclusive results [32,33,34]. A systematic review and meta-analysis of cohort studies reported a 5% lower risk of incident hypertension for each 100 mg/day higher magnesium intake [35]. Two meta-analyses of randomized controlled trials (RCTs) found that participants receiving magnesium supplements had a significant reduction in blood pressure vs. controls [36,37]. Former meta-analyses suggested benefits with less prominent effects, possibly due to heterogeneity of included studies [38,39].

Previous large long-term prospective studies evaluating the relationship of dietary magnesium with hypertension have been conducted mainly in the United States [35], but none in Mediterranean populations. Because overweight and obesity are major risk factors for the development of hypertension [40], we aimed to prospectively assess the association of magnesium intake with the risk of incident hypertension in relation to the presence of overweight and obesity in the Mediterranean population of the SUN (“Seguimiento Universidad de Navarra”) longitudinal project.

## 2. Methods

### 2.1. Study Design and Population

The SUN Project is a prospective, longitudinal, multipurpose, and permanently open cohort study of university graduates started in 1999, updated every 2 years with information on lifestyle, diet, risk factors, and medical conditions during follow-up, as previously described in detail [41,42]. For the present analyses, we considered the last available database as of 1 December, 2016, corresponding to 22,560 enrolled participants. We excluded (a) 4404 who had prevalent hypertension at baseline; (b) 260 who answered the baseline questionnaire after 1 March, 2014, not having spent sufficient time in the study as to be able to fill and return the first follow-up questionnaire (<2 years plus 9 months considering the time interval to return the questionnaires); (c) 1721 participants who reported total energy intake outside of predefined limits (<500 or >3500 kcal/day for women and <800 or >4000 kcal/day for men) [43]; (d) 753 with other chronic diseases at baseline (diabetes, cardiovascular disease, or cancer); and (e) 1365 participants who were lost to follow up. The final analytic population included 14,057 participants (Figure S1 Supplementary Materials). The overall retention rate was 91.2% (91.2% of participants recruited at least 2 years and 9 months ago returned ≥1 of the follow-up questionnaires).

### 2.2. Ethics

Participants received written information on their specific data to be requested by future questionnaires, the future feedback from the research team, and the protection to safeguard their privacy. We also informed all potential candidates of their right to refuse to participate in the SUN study or to withdraw their consent to participate at any time without retaliation, according to the principles of the Declaration of Helsinki. The voluntary completion of the baseline questionnaire was considered to imply informed consent. The Research Ethics Committee of the University of Navarra approved this method to request the informed consent of participants (Project identification code 2001_30).

### 2.3. Dietary and Exposure Assessment

The details of the dietary assessment with a validated semi-quantitative 136-item food frequency questionnaire (FFQ) in the SUN Project at baseline has been previously described [44]. The content of nutrients in the FFQ was calculated as previously described [44,45,46] by means of the latest available Spanish food composition tables [47,48]. The FFQ also contained additional questions about vitamin and mineral supplements, such as magnesium [41]. The validity [44,45] and reproducibility [46] of the FFQ has been previously reported. The last validation study showed a high intra-class correlation coefficient (ICC) between the questionnaire and the reference method (four 3-day dietary records) for dietary components (ICC = 0.66 and ICC = 0.75 after energy adjustment; both *p* < 0.001). The reproducibility study showed similarly high ICCs, with corresponding values of 0.86 and 0.88, respectively [45]. Dietary magnesium was calculated from magnesium-rich foods assessed with a 136-item FFQ. Total magnesium intake was computed as the sum of dietary magnesium and magnesium intake derived from supplements [44]. Nutrient scores were calculated as frequency multiplied by nutrient composition of specified portion size where frequencies were measured in 9 categories from never/almost never to >6 servings/day for each food item. Nutrient intake scores were computed using ad hoc computer software specifically developed for this aim. A trained dietician updated the nutrient data bank using the latest available information included in food composition tables for Spain [47,48]. A substudy in our cohort showed good reproducibility for assessing magnesium intake; the Pearson correlation coefficients were 0.69 (<1 y between responses) and 0.65 (≥1 y between responses) [46]. Adherence to the Mediterranean food pattern was assessed using the 9-point score proposed by Trichopoulou et al. [49]. One point was assigned to persons whose consumption was higher or equal to the sex-specific median of components with most likely beneficial effects (vegetables, fruits/nuts, legumes, fish/seafood, cereals, and monounsaturated/saturated fatty acid [MUFA/SFA] ratio), otherwise no point was assigned; no point was assigned to persons with consumption higher or equal to the sex-specific median of components proven not to be beneficial (meat and dairy products), otherwise one point was assigned. For ethanol, one point was assigned to men consuming 10–50 g/day and to women consuming 5–25 g/day, otherwise no point was assigned.

### 2.4. Ascertainment of Incident Hypertension

The main outcome was defined by a medical diagnosis or self-report of hypertension in any follow-up questionnaire, which has been formerly repeatedly validated [50,51,52]. In one of the validation substudies nested in the SUN cohort a subsample of the SUN cohort participants who indicated a medical diagnosis of hypertension, were on antihypertensive medication, or both in any of the follow-up questionnaires, two physicians (blinded to the information reported by participants) did direct measurements of blood pressure in the homes of participants who confirmed self-reported hypertension or self-reported hypertension-free status in a subsample of the cohort. Considering blood pressure >140/90 mmHg as confirmed by the 2018 European guidelines [53], 82.3% (95% confidence interval (CI) 72.8, 92.8%) of self-reported hypertension in the questionnaires was confirmed. Among those who did not report a diagnosis of hypertension in the questionnaires, 85.4% (95% CI 72.4, 89.1%) were confirmed as non-hypertensives [50].

For the present study, participants with self-reported hypertension but missing information on the date of diagnosis, the mid-point between the date of completion of the follow-up questionnaire with the self-reported medical diagnosis of hypertension and the previous follow-up questionnaire were used to impute the date of diagnosis.

### 2.5. Other Covariates

We considered the following additional covariates in the statistical models: socio-demographic characteristics (age as a continuous variable and in 10 categories, sex, marital status), anthropometric parameters (body weight, height, body mass index (BMI)), lifestyle risk factors (physical activity, smoking habit in three categories, alcohol use, hours/day spent watching television, following special diets, total energy intake, snacking between meals, sugar-sweetened beverage consumption, years of university education), and other confounding risk factors (borderline hypertension at baseline, family history of hypertension, sodium intake, potassium intake, calcium intake, fiber intake, analgesic consumption, diuretic use). We assessed physical activity with a questionnaire formerly validated with objective measurements using a triaxial accelerometer (RT3 Triaxial Research Tracker) as reference (Spearman correlation coefficient of 0.51; *p* < 0.001) [54]. The magnitude of physical activity was expressed in metabolic equivalent tasks (METs-h/week) according to the calculation of the time spent at each activity in hours/week multiplied by its typical energy expenditure [55]. Weight and BMI reported by each participant were previously validated in a subsample of the SUN cohort [56].

### 2.6. Statistical Analysis

For the description of the baseline characteristics of the sample, we computed means and SDs for continuous variables and proportions for categorical variables across categories of daily dietary magnesium intake (<200 mg, 200–500 mg, >500 mg). We chose to use these exact boundaries instead of quantiles because the groups thus built were more meaningful per se and could be more easily used for future comparisons with similar studies. This is in line with current recommendations given in epidemiology about procedures to categorize continuous variables [57]. After a forward stepwise selection algorithm we used nested regression models to evaluate the contribution of the main sources of dietary magnesium intake to total dietary magnesium intake (Table 1).

We calculated the follow-up time for each participant from the date of returning the baseline questionnaire to the date of the diagnosis of incident hypertension or to the date of returning the last follow-up questionnaire. We computed incident hypertension rates across baseline categories of daily dietary magnesium intake, and calculated HRs and 95% CI by means of Cox proportional hazards models using the lowest category of magnesium intake as the reference. To test the proportional-hazards assumption we used the test based on Schoenfeld residuals after fitting the model. Age was the underlying time variable, and we performed various adjustments as follows: First, hazard ratios (HRs) were adjusted for age (10 categories used as stratification variables in the Cox model, in addition to using age as the time variable) and sex; second, HRs were additionally adjusted for BMI (in 5 categories), total energy intake (continuous), following special diets at baseline, physical activity (METs-h/week), alcohol (g/d, continuous), and smoking (3 categories); third, HRs were additionally adjusted for marital status (2 categories), body weight changes, years of university education, borderline hypertension at baseline, family history of hypertension, and year of entrance to the cohort; fourth, HRs were additionally adjusted for sodium intake, potassium intake, calcium intake, fiber intake, hours per day spent watching television (continuous), analgesic consumption, diuretic use, and sugar-sweetened beverage consumption. We used dietary magnesium intake as a continuous variable to calculate the significance of the linear trend tests. We used multivariable-adjusted HR estimates to calculate dose–response associations between total dietary magnesium intake and incident hypertension. We performed various sensitivity analyses estimating the fully adjusted HRs for total dietary magnesium and incident hypertension after changing several assumptions: (1) including only men or only women, (2) adopting allowed limits for total energy intake from percentile 1 to 99, (3) censoring the follow-up time of participants at 6 or 8 years, (4) excluding participants with early incident hypertension (during the first 2 years), (5) adjusting for the Mediterranean diet (MeDiet) score [49], (6) adjusting for dietary fiber intake, and (7) adjusting for diuretic use. We also estimated the Pearson’s correlation coefficient between dietary magnesium intake and both adherence to the traditional Mediterranean diet and dietary fiber intake. We performed these analyses with Stata software package version 12 (Stata Corp). All *p* values were 2-tailed and significance was set at *p* < 0.05. Values in the text are means ± SDs unless otherwise indicated.

## 3. Results

### 3.1. Characteristics of Participants

Among 135,361 person—years of follow-up (mean follow-up: 9.6 years; range: 0.05–17.1 years) during the 1999–2016 period we observed 1406 cases of incident hypertension in the SUN cohort. Table 2 displays the baseline characteristics of participants, comprising anthropometric, demographic, and lifestyle features as well as food consumption and nutrient intake, according to magnesium intake per day in three categories. Participants with magnesium intake < 200 mg/d vs. those with >500 mg/d intake were more likely to be men, single women, married men, current smokers, those with a former history of depression or hypercholesterolemia, those with lower levels of leisure-time physical activity, and those with a lower frequency of between-meal snacking. They were also more likely to have lower scores of adherence to the MeDiet, less consumption of vegetables, fruit, legumes, cereals, whole cereals, nuts, olive oil, eggs, fish, whole and low-fat dairy, meat, coffee, total energy intake, and alcohol. They were more likely to have higher intakes of total fat and mono and saturated fatty acids, and lower intakes of vitamins C and D, iron from heme sources, folate, sodium, potassium, calcium, and dietary fiber.

### 3.2. Magnesium Intake, Incident Hypertension, and Obesity

**Figure 1:**
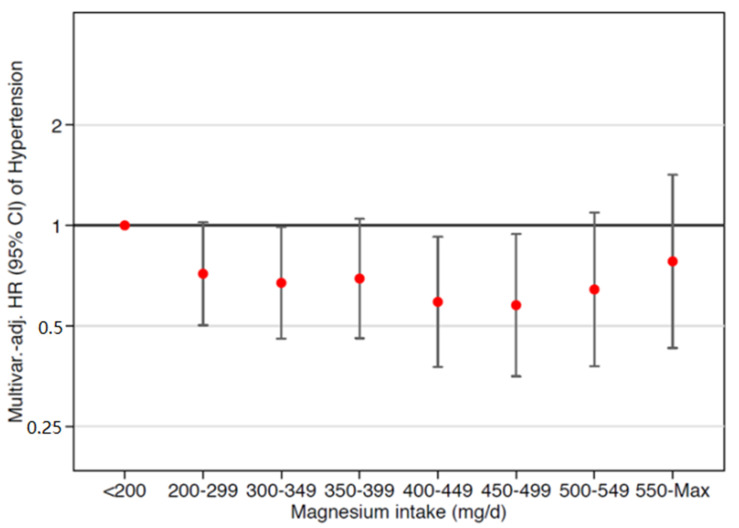
Multivariable-adjusted hazard ratios for incident hypertension by categories of magnesium intake (from <200 mg/d to >550 mg/d). The model was adjusted for age, sex, BMI, total energy intake, following special diets at baseline, physical activity, alcohol consumption, smoking, marital status, body weight changes, years of university education, borderline hypertension at baseline, family history of hypertension, year of entrance to the cohort, sodium intake, potassium intake, calcium intake, hours per day spent watching television, analgesic consumption, and sugar-sweetened beverage consumption.

As shown in Table 3, Table S1 (Supplementary Materials), and Figure 1, a significant inverse association in the Cox models was observed for progressively higher magnesium intake up to 500 mg/d after adjustments for sex and age, which remained significant after multivariate adjustments (Models 2, 3, and 4) vs. an intake < 200 mg/d (reference). This association was significant only for Model 3 for magnesium intakes > 500 mg/d (Table 3). Considering diverse categories of BMI and of magnesium intake, the risk of developing hypertension was still higher for participants with a magnesium intake < 200 mg/d and BMI 23–27 kg/m^2^ and even greater for those with a BMI > 27 kg/m^2^ vs. participants with a BMI < 23 kg/m^2^ (Figure S2, Supplementary Materials).

**Figure 2:**
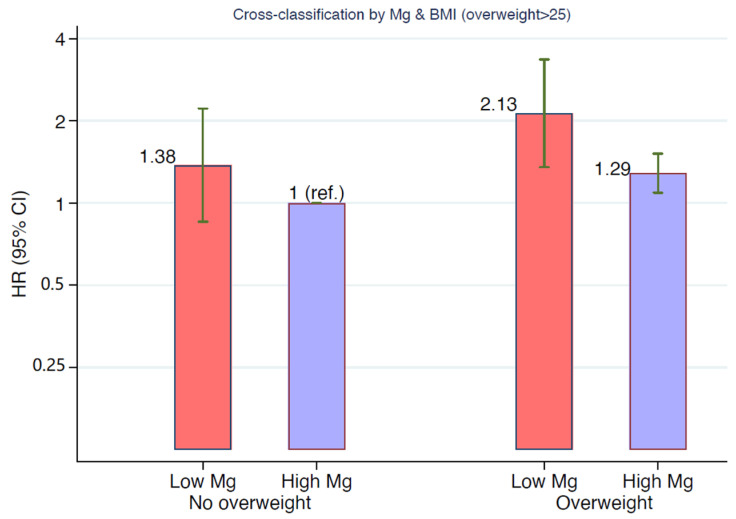
Multivariate-adjusted HR of incident hypertension according to cross-classification by BMI (lower and higher than 25 kg/m^2^) and magnesium intake (lower and higher than 200 mg/d) in participants from the SUN Project. The model was adjusted for age, sex, BMI, total energy intake, following special diets at baseline, physical activity, alcohol consumption, smoking, marital status, body weight changes, years of university education, borderline hypertension at baseline, family history of hypertension, year of entrance to the cohort, sodium intake, potassium intake, calcium intake, hours per day spent watching television, analgesic consumption, and sugar-sweetened beverage consumption.

Taking into consideration participants with a magnesium intake less or greater than 200 mg/d and BMI less or greater than 25 kg/m^2^, the multivariate-adjusted risk of incident hypertension was double for the latter with a low magnesium intake (Figure 2). Lean participants (BMI < 25 kg/m^2^) with low magnesium intake (<200 mg/d) had a similar risk as those who were overweight or obese with high magnesium intake (>200 mg/d) (Figure 2). When we assessed the potential modifying effect of magnesium by BMI, we did not find any statistically significant interaction (*p* = 0.12) with a four-degree of freedom likelihood ratio test using magnesium in three categories (<200, 200–500, >500 mg/d) and BMI in three categories (<23, 23–27, >27 kg/m^2^).

### 3.3. Magnesium Intake, Incident Hypertension, and Mediterranean Diet

**Figure 3:**
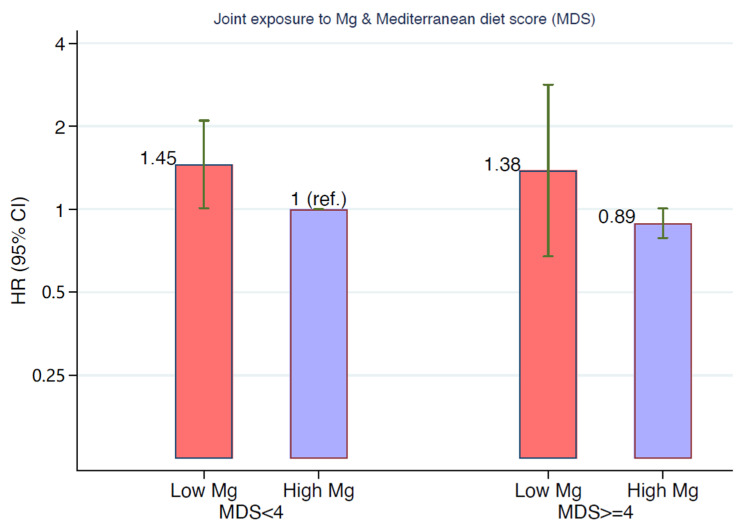
Multivariable-adjusted hazard ratios for incident hypertension. Joint exposure to Mediterranean diet score (lower and higher than four points) and magnesium intake (lower and higher than 200 mg/d). The model was adjusted for age, sex, BMI, total energy intake, following special diets at baseline, physical activity, alcohol consumption, smoking, marital status, body weight changes, years of university education, borderline hypertension at baseline, family history of hypertension, year of entrance to the cohort, sodium intake, potassium intake, calcium intake, hours per day spent watching television, analgesic consumption, and sugar-sweetened beverage consumption.

We found a positive association of magnesium intake with the MeDiet adherence score (r = 0.46; *p* < 0.0001). However, low magnesium intake was similarly associated with ~50% relative increase in the risk of incident hypertension across categories of MeDiet adherence. Within strata of low (<4) and high (≥4) scores of adherence to the MeDiet, the HRs for the association between low magnesium and incident hypertension were similar, with values of HR = 1.45 and HR = 1.38, respectively (Figure 3).

### 3.4. Sensitivity Analyses

We performed several sensitivity analyses for the significant multivariate-adjusted inverse association of dietary magnesium intake and incident hypertension (Table 4). This association remained significant in the different scenarios that we considered: after restricting the analyses only to women, censoring the follow-up time of participants at six or eight years, excluding participants with early incident hypertension (first two years), and after additional adjustment for the score of adherence to the MeDiet, dietary fiber intake, and diuretic use. Supplements of magnesium were used in only 8.5% of participants, with a very low mean dose in those taking them (40.8 ± 50.7 mg/d). When we excluded participants who consumed magnesium supplements the results were not modified.

## 4. Discussion

The present analyses on data from the SUN Project, a prospective, well-characterized, and large cohort of Spanish university graduates, indicated that dietary magnesium intake was inversely and significantly associated with the incidence of hypertension after a long-term follow-up, independently of a number of potential confounders considered in the fully adjusted statistical models. The risk of incident hypertension in this population associated with low magnesium intake (<200 mg/d) was independent of adherence to the MeDiet. Participants who were overweight and obese with magnesium intakes below 200 mg/d had a further significantly excess risk compared to lean and overweight or obese participants with higher magnesium intake.

Previous studies assessing the relationship of dietary and supplemental magnesium with the incidence of hypertension and diverse meta-analyses on cohort studies and RCTs have reported protective effects [35,36,37,39]. A recent study examining meta-analyses on the effects of electrolytes on hypertension showed that magnesium intake had the greatest benefit, followed by potassium intake and by salt reduction [58]. As in our study, previous studies evaluating the relationship of dietary magnesium and incident hypertension used as reference a magnesium intake < 200 mg/day [27,28,29,35]. This consistent decision appears to be based on a sound biological plausibility for assuming a deleterious effect of magnesium intakes below 200 mg/d. Thus, previous evidence is in agreement with our results of an inverse relationship of low magnesium intake and incident hypertension, for the first time in a Mediterranean population.

Several mechanisms can explain the protective effects of a diet rich in magnesium against hypertension, including its calcium antagonist actions and its effects on endothelial function, vascular tone, reactivity, and vascular cell growth [6,7,8,10,11,13,14,59]. Through these actions magnesium has modulatory effects on blood pressure. Indeed, modifications in magnesium status lead to alterations in vascular tone and, consequently, to fluctuations in arterial blood pressure and reactivity, especially because of its calcium-antagonist properties [7,8]. Magnesium deficiency stimulates angiotensin II-mediated aldosterone production and the synthesis of thromboxane and prostaglandins with vasoconstrictor effects [9]. Besides the direct actions of magnesium on vascular smooth muscle cells, it also has modulatory actions on endothelial function, which in turn contributes to its vasodilatory effects. In normal conditions, endothelium plays a key role in the regulation of the vasomotor tone by synthesizing vasodilatory prostacyclins and nitric oxide. Magnesium has been shown to improve endothelial function in humans [10,11] and to be associated with circulating markers of systemic inflammation and endothelial dysfunction in women [12]. Magnesium was shown to participate in vascular structure and remodeling [13], whereas magnesium-signaling increased endothelial survival, adhesion, migration, and growth [59].

We observed that the deleterious effects of a magnesium intake below 200 mg/d on incident hypertension were dramatically worse in overweight and obese participants. Obesity is a well-recognized risk factor for hypertension [1,3,4]. In particular, excessive visceral fat distribution is associated with hormonal, inflammatory, and endothelial alterations, which increase hypertension risk and cardiovascular morbidity and mortality. It is widely accepted that BMIs corresponding to the WHO classification of overweight (>25 kg/m^2^) or obese (>30 kg/m^2^) are related to an increased risk of incident hypertension. However, these categories include several levels of BMI and do not allow us to perceive in detail the linear association of overweight/obesity and incident hypertension. There is evidence in several studies and populations of a significant and linear increase in blood pressure and incident hypertension for BMI values greater than 22 kg/m^2^ [60,61,62]. Even at values considered “normal” by the WHO classification (20–25 kg/m^2^), those persons with a BMI greater than 22–23 kg/m^2^ have a significantly higher risk of incident hypertension compared to those with lower BMI values. In the present study, we observed that in persons with a BMI 23–27 kg/m^2^ and more so in those with a BMI > 27 kg/m^2^, the risk of hypertension was multiplied if they had a magnesium-deficient diet, which might add another pathophysiological key mediator linked to a magnesium deficit.

Interestingly, participants with a BMI < 25 kg/m^2^ and magnesium intake < 200 mg/d had a similar risk of developing hypertension as those with a BMI > 25 kg/m^2^ with an adequate magnesium intake (Figure 2). This could correspond to metabolically obese normal-weight (MONW) participants, described since 1981 [63,64]. These patients with a BMI < 25 kg/m^2^ have metabolic abnormalities more commonly observed in obese patients, such as abdominal fat distribution, insulin resistance, hypertriglyceridemia, and high blood pressure. Although the definition of MONW patients has not been univocal, some metabolic (i.e., triglyceride index) [65] and genetic markers [66] have been described in order to identify these high-risk patients. This condition has been associated with low concentrations of serum magnesium [67], and improvements in blood pressure, fasting glucose, and triglyceride concentrations after oral magnesium administration have been reported in MONW persons [68].

Furthermore, participants with a BMI > 25 kg/m^2^ and adequate magnesium intake had lower risk of incident hypertension vs. overweight or obese participants with low magnesium intake, reinforcing the protective effect of dietary magnesium intake against hypertension even in obese persons. This may suggest the protective effect of dietary magnesium intake against obesity-associated hypertension, and that insufficient dietary magnesium intake is associated with increased risk of hypertension in all, regardless of weight.

The positive relationship of dietary magnesium intake with the MeDiet adherence score in our cohort (r = 0.46; *p* < 0.0001) may lead one to think that the overall dietary quality may account for the protection apparently afforded by magnesium in our results. However, we observed that the inverse association of dietary magnesium with incident hypertension was similar regardless of adherence to the MeDiet. Most, but not all, MeDiet components contain magnesium; our results might indicate that those components rich in magnesium content (i.e., vegetables, nuts, whole cereals, legumes) were particularly protective even when other points of the MeDiet adherence score were not met. Participants with low conformity to this dietary pattern with an adequate magnesium intake were protected, as were obese participants with an adequate magnesium intake. Of note, nearly all magnesium intake in our cohort was from dietary sources, and the use of magnesium supplements was marginal and at low doses.

We observed a strong correlation between dietary magnesium and fiber intakes (r = 0.87; *p* < 0.0001). This has been reported in previous studies [69,70,71,72] and is related to the fact that both nutrients are contained in the same type of foods. Furthermore, in some studies the association of dietary magnesium intake with metabolic outcomes was attenuated when the results were adjusted for dietary fiber [70,71,72].

Regarding the association of dietary fiber intake and incident hypertension, the results are not yet completely conclusive [73]. Interestingly, the association that we observed between a low magnesium intake and incident hypertension remained significant after adjusting for dietary fiber intake in the sensitivity analyses (Table 4).

It should be also considered that the absorption, hence, bioavailability of magnesium is reduced with high amounts of fiber intake [74], such as what we observed in the category of higher magnesium intake (>500 mg/d), where the significance of the association with incident hypertension was marginal.

Currently, nutrition research underscores the relevance of studying food and nutrient combinations, as opposed to the reductionist approach based on single nutrients or foods widely used in the past. Dietary patterns reported to significantly reduce blood pressure in hypertensive and pre-hypertensive patients include Dietary Approaches to Stop Hypertension (DASH) and the MeDiet [75]. Interestingly, DASH, ranked as the most effective dietary model in reducing blood pressure [75], emphasizes the high content of minerals, including magnesium. Nevertheless, it is unlikely that magnesium alone is responsible for the hypertension preventive effects of these dietary patterns, but it is confidently an important independent determinant, remaining significant after multivariate adjustments and regardless high or low conformity with the MeDiet. It should be remembered that foods rich in magnesium are also rich in other components with recognized health benefits. The independent effects of magnesium and the detrimental consequences of a magnesium-poor diet even in lean persons may identify a missing piece of the puzzle in order to help explain mechanistically why a diet rich in vegetables, fruit, nuts, whole cereals, and legumes has anti-hypertensive and cardiovascular benefits. Thus, magnesium seems to have a strong protective effect against hypertension in a world where the Western diet, rich in ultra-processed foods and poor in magnesium, is frequently followed [19,20,21,22], but we admit that magnesium likely acts in conjunction and harmony with other nutrients present in the same food sources as magnesium.

Among the strengths of the present study we include the large sample size, the prospective design, the extensive follow-up, the high retention rate, and the possibility to adjust for numerous confounding factors. Potential limitations include that, regardless of the SUN cohort characteristic, the application of our results in other contexts must be based on common biological mechanisms and not on simple statistical “representativeness.” Thus, we used restriction to reduce potential confounding factors, such as socioeconomic status, education, disease, and expected access to health care. Future studies are needed in order to test our findings in other populations. Another limitation could be the use of self-reported information; however, self-reported parameters such as hypertension, weight and BMI have been previously validated [50,51,52,56]. In the fully adjusted multivariate analyses, when dietary magnesium beyond 500 mg/day is not significantly associated with incident hypertension it might be due to the lower number of participants in that group and the consequent reduction in statistical power. Furthermore, although the size of the group may be similar to the one that consumed 200–300 mg of magnesium per day and was significant, the confounders added in the fully adjusted multivariate analysis increased the standard error in the >500 mg/day group, perhaps because it was the extreme group, which had more different values in the confounders. However, all point estimates suggest an inverse association with the association being significant in one of the multivariate adjusted models (Model 3). For some other confounding factors such as phytate, long-chain triglycerides, proton pump inhibitors, and glomerular filtration rate, we did not have enough information to include them in the analyses. The use of a validated FFQ in a prospective cohort does not fully preclude the possibility of non-differential information bias, but we found sensible correlation coefficients for the exposures. Because the potential measurement error in the assessment of diet is expected to be non-differential, it would likely bias our results towards the null value. FFQ remains a useful and inexpensive method to characterize dietary habits of large samples followed over extended periods of time, aiming to assess associations with incident clinical outcomes [76]. Another limitation is the possible change in customary diet, and thus in magnesium status, during follow-up.

## 5. Future Lines of Research

Based on our present results in a Mediterranean cohort and those of previous studies from other regions cited above, the populations that currently tend towards a diet poor in magnesium can benefit from correcting this deficit through the introduction and sufficient use of magnesium-rich foods in their usual diets. 

Furthermore, the benefit is multiplied by the fact that these same foods contain other substances that are beneficial to health.

It is clear that people with a higher cardiovascular risk, including those with a higher risk for hypertension, would benefit the most. Nevertheless, more evidence is needed from other populations, e.g., from Asia, South America, Africa, and other European countries, which may help in further developing healthy dietary recommendations and interventions.

## 6. Conclusions

A dietary pattern with magnesium intake below 200 mg/d was strongly and independently associated with a higher risk of developing hypertension in a relatively young Mediterranean cohort. This association was worse for overweight and obese participants and independent of the conformity with the Mediterranean dietary pattern. These results emphasize the importance of controlling overweight and to broadly encourage the consumption of foods such as vegetables, nuts, whole cereals, and legumes, optimal dietary sources of magnesium, in order to prevent hypertension, the main risk factor for cardiovascular and cerebrovascular events in all populations.

## Figures and Tables

**Table 1:** Main sources of variability (cumulative R^2^) and main sources (%) of dietary magnesium intake among participants in the SUN (“Seguimiento Universidad de Navarra”) cohort, 1999–2016.

Dietary Magnesium Source	% of Magnesium Intake	Cumulative R^2^
Vegetables	27.0	0.36
Fruit	13.0	0.50
Dairy	15.0	0.63
Nuts	3.7	0.69
Legumes	6.9	0.73
Fish and seafood	7.3	0.85

**Vegetables:** carrot, Swiss chard, cabbage, lettuce, tomatoes, green beans, eggplant, peppers, asparagus, other fresh vegetables. **Fruits:** Citrus, banana, apple, pear, strawberry, peach, cherry, fig, melon, watermelon, grapes, kiwi, mango, canned fruit. **Dairy:** milk (whole, low-fat, condensed, milkshake, cream), yogurt (whole, low-fat), cheese (soft, hard, fresh, old, processed, curd), flan, ice cream. **Nuts:** almonds, peanuts, hazelnuts, pistachios, pine nuts, walnuts. **Legumes:** lentils, chickpeas, beans, peas. **Fish and Seafood:** fish (white, blue, processed), Seafood (oysters, prawns, octopus).

**Table 2:** Baseline characteristics of participants according to three categories of magnesium intake among participants in the SUN (“Seguimiento Universidad de Navarra”) cohort, 1999–2016 ^a^.

	Magnesium Intake (mg/d)		
	<200	200–500	>500
Mg intake (mg/d)	155.1 ± 42.5	370.8 ± 72.8	588.7 ± 85.6
N	267	10782	3008
Women, %	63.7	66.6	68.7
Age, y	37.0 (11.3)	34.8 (10.5)	36.7 (11.2)
Married women, %	44.6	45.8	47.3
Married men, %	55.4	54.2	52.7
University education, y	5.1	5.0	5.0
BMI, kg/m^2^	23.5 (3.4)	23.0 (3.2)	22.9 (3.1)
Smoking			
Current, %	33.3	27.3	21.7
Former smoker, %	18.7	21.5	23.5
Alcohol (g/d)	3.32 (5.21)	5.8 (8.48)	6.38 (9.75)
Leisure-time physical activity, METs-h/week	19.8 (21.4)	25.4 (21.7)	32.2 (28.6)
Television watching, h/d	1.6 (1.1)	1.6 (1.2)	1.6 (1.2)
History of depression at baseline, %	16.1	10.5	11.5
Hypercholesterolemia at baseline, %	16.9	13.0	14.1
Total energy intake, kcal/d	1110 (290)	2216 (518)	2947 (450)
Adoption of special diets, %	8.3	6.8	8.1
Between-meal snacking, %	28.2	34.5	64.7
**Dietary consumption**			
Mediterranean diet score ^b^	2.49 ± 1.1	3.74 ± 1.7	5.22 ± 1.6
Vegetables (g/d)	146 (137)	452 (229)	820 (465)
Fruit (g/d)	88 (109)	282 (203)	573 (420)
Legumes (g/d)	8.5 (10.2)	21 (13)	31 (28)
Cereals (g/d)	33 (38)	96 (66)	127 (80)
Whole bread (g/d)	1.2 (4.7)	1.2 (4.7)	29 (49)
Nuts (g/d)	1.6 (2.9)	5.4 (7.6)	14 (19)
Olive oil (g/d)	8.8 (12.3)	15 (13)	18 (14)
Eggs (g/d)	14 (15)	23 (15)	25 (16)
Fish and other seafood (g/d)	49 (42)	88 (50)	129 (77)
Whole dairy products (g/d)	106 (139)	197 (190)	224 (232)
Low-fat dairy products (g/d)	82 (121)	203 (218)	324 (315)
Meat (g/d)	100 (69)	173 (75)	194 (89)
Coffee (cups/d)	3.3 (2.6)	3.8 (2.4)	3.9 (2.5)
Sugar-sweetened beverages (servings/d) ^c^	0.20 (0.5)	0.21 (0.4)	0.19 (0.4)
**Dietary intake**			
Carbohydrates (% of energy)	39 (13)	43 (7.0)	46 (7.2)
Protein (% of energy)	18 (7.4)	18 (3.2)	18 (3.1)
Total fat (% of energy)	41 (10)	37 (6.3)	34 (6.3)
MUFAs (% of energy)	18 (6.4)	16 (3.6)	15 (3.4)
SFAs (% of energy)	15 (5.4)	13 (3)	11 (3.1)
PUFAs (% of energy)	5.5 (2.5)	5.2 (1.5)	5.0 (1.5)
Vitamin C (mg/d)	85 (72)	241 (113)	435 (193)
Vitamin D (mcg/d)	1.9 (1.7)	3.4 (2.1)	4.9 (3.2)
Iron from heme sources (mg/d)	6.7 (2.3)	15 (3.3)	23 (4.4)
Folate (mcg/d)	136 (71)	356 (111)	615 (197)
Na intake (mg/d)	2129 ± 1459	3785 ± 2112	4666 ± 2828
K intake (g/d)	1720 ± 612	4245 ± 962	6823 ± 1442
Ca intake (g/d)	502 ± 245	1129 ± 368	1651 ± 505
Dietary fiber (g/1000 kcal/d)	8.3 (4.8)	24 (7.4)	43 (13)

BMI: body mass index; MET: metabolic equivalent task; MUFA: monounsaturated fatty acid; SFA: saturated fatty acid; PUFA: polyunsaturated fatty acid. ^a^ Values are mean (SD) unless otherwise stated. ^b^ The Mediterranean score was calculated as proposed by Trichopoulou et al. [49]. ^c^ One serving of sugar-sweetened beverages = 200 mL.

**Table 3:** Association between magnesium intake and incident hypertension among participants in the SUN (“Seguimiento Universidad de Navarra”) cohort, 1999–2016 ^a^.

Dietary Magnesium	Categories of Daily Magnesium Intake (mg/day)					
	<200	200–300	300–400	400–500	>500	*p*-Trend
n	267	2051	4659	4072	3008	
Incident hypertension (n)	43	212	459	354	338	
Person—years	2698	19541	45294	39469	28359	
Median (g/d)	168.9	267.4	353.0	444.5	536.5	
Crude incident hypertension rate (×10^−3^)	1.59	1.08	1.10	0.90	1.12	
Age- and sex-adjusted HR Model 1	1 (ref.)	0.77 (0.56, 1.07)	0.73 (0.53, 0.99)	0.64 (0.46, 0.87)	0.78 (0.57, 1.07)	0.455
Multivariate-adjusted HR ^b^ Model 2	1 (ref.)	0.69 (0.49, 0.97)	0.66 (0.47, 0.94)	0.57 (0.39, 0.83)	0.69 (0.45, 1.04)	0.618
Multivariate-adjusted HR ^c^ Model 3	1 (ref.)	0.66 (0.47, 0.93)	0.64 (0.45, 0.90)	0.54 (0.37, 0.80)	0.66 (0.43, 0.99)	0.589
Multivariate-adjusted HR ^d^ Model 4	1 (ref.)	0.67 (0.47, 0.96)	0.65 (0.45, 0.95)	0.55 (0.36, 0.84)	0.66 (0.39, 1.10)	0.470

SUN, Seguimiento Universidad de Navarra. ^a^ Values are HR estimated with the Cox regression and 95% confidence intervals (CI). ^b^ Model 2: HR adjusted for age (10 categories), sex, body mass index (in five categories), total energy intake, following special diets at baseline, physical activity (METs-h/week), alcohol (g/d), and smoking (three categories). ^c^ Model 3: HR adjusted for factors in Model 2 plus marital status, body weight changes, years of university education, borderline hypertension at baseline, family history of hypertension, and year of entrance to the cohort. ^d^ Model 4: HR adjusted for factors in Model 3 plus sodium intake, potassium intake, calcium intake, hours per day spent watching television, analgesic consumption, and sugar-sweetened beverage consumption.

**Table 4:** Sensitivity analyses: multivariable-adjusted hazard ratios of incident hypertension associated with low dietary magnesium intake (<200 mg/d) among participants in the SUN Project, 1999–2016) ^a^.

	n	Incident Hypertension n	HR (95% CI) ^b^
Main analysis	14,057	1406	1.51 (1.08, 2.11)
Including only women	9416	676	1.77 (1.10, 2.87)
Changing allowable energy limits (percentiles 1–99) ^c^	14,944	1473	1.20 (0.70, 2.05)
Censoring follow-up at 8 y	9416	446	2.33 (1.25, 4.36)
Censoring follow-up at 6 y	9416	365	2.42 (1.12, 5.23)
Excluding early incident hypertension (first 2 y)	13,766	1210	1.54 (1.07, 2.21)
Adjusting for the Mediterranean diet score ^d^	14,057	1406	1.48 (1.06, 2.06)
Adjusting for fiber intake	14,057	1406	1.48 (1.06, 2.06)
Adjusting for fiber intake below the median	14,057	1406	1.76 (1.01, 3.09)
Adjusting for use of diuretics	14,057	1406	1.48 (1.06, 2.07)

SUN, Seguimiento Universidad de Navarra. ^a^ Values are HR estimated with Cox regression and 95% CI. If the CI includes 1.00, the results are not significant; *p* > 0.05 (two-tailed). ^b^ HR adjusted for age (10 categories), sex, BMI (in five categories), total energy intake, year of entering the cohort (four categories), years of university education, smoking (three categories), borderline hypertension at baseline, family history of hypertension, marital status, alcohol (g/d), physical activity (METs-h/week), body weight changes, hours per day spent watching television, following special diets at baseline, snacking between meals, sodium intake, potassium intake, calcium intake, analgesic consumption, and sugar-sweetened beverage consumption. ^c^ The criteria for exclusion here were only based on energy intake, but not on the number of blank items. This is the reason why the number of participants was higher in this ancillary analysis. ^d^ The Mediterranean score was calculated as proposed by Trichopoulou et al. [49].

## Data Availability

The data that support the findings of this study are available from the SUN Project at sun@unav.es, upon reasonable request.

## Supplementary Materials

The following are available online at https://www.mdpi.com/2072-6643/13/1/125/s1. Table S1. Association between magnesium intake and incident hypertension among participants in the SUN (“Seguimiento Universidad de Navarra”) cohort, 1999–2016; Figure S1. Flow chart depicting the selection process among participants of the SUN project to be included in the present analyses; Figure S2. Multivariate-adjusted HR of incident hypertension according to cross-classification by BMI in three categories and magnesium intake in four categories in participants from the SUN project. The model was adjusted for age, sex, BMI, total energy intake, following special diets at baseline, physical activity, alcohol consumption, smoking, marital status, body weight changes, years of university education, borderline hypertension at baseline, family history of hypertension, year of entrance to the cohort, sodium intake, potassium intake, calcium intake, hours per day spent watching television, analgesic consumption, and sugar-sweetened beverages consumption.
