# Supplementary material for: Low Dietary Magnesium and Overweight/Obesity in a Mediterranean Population: A Detrimental Synergy for the Development of Hypertension. The SUN Project
Source: Nutrients. 2020 Dec 31;13(1):125. doi: 10.3390/nu13010125 (PMC7824180; doi:10.3390/nu13010125)
Supplement: Supplementary file 1 [file nutrients-13-00125-s001.pdf]

Table S1. Association between magnesium intake and incident hypertension among participants in the SUN ("Seguimiento Universidad de Navarra") cohort, 1999-2016[a]

Dietary Magnesium	Categories of Daily Magnesium Intake (mg/day)							
	**<200**	**200-300**	**300-400**	**400-500**	**500-600**	**600-700**	**>700**	***P-trend***
n	267	2051	4659	4072	1990	732	286	
Incident hypertension (n)	43	212	459	354	213	89	36	
Person-years	2698	19541	45294	39469	19013	6876	2471	
Median (g/d)	168.9	267.4	353.0	444.5	538.0	636.6	757.4	
Crude rate (x10^{-3})	1.59	1.08	1.10	0.89	1.12	1.29	1.46	
Age-, sex-adjusted HR Model 1	1 (ref.)	**0.77 (0.56, 1.07)**	**0.73 (0.53, 0.99)**	**0.64 (0.46, 0.87)**	0.76 (0.55, 1.05)	0.82 (0.57, 1.18)	0.83 (0.53, 1.29)	0.46
Multivariate-adjusted HR[b] Model 2	1 (ref.)	**0.69 (0.49, 0.98)**	**0.67 (0.47, 0.95)**	**0.58 (0.39, 0.84)**	0.67 (0.44, 1.02)	0.77 (0.49, 1.24)	0.74 (0.43, 1.28)	0.62
Multivariate-adjusted HR[c] Model 3	1 (ref.)	**0.66 (0.47, 0.94)**	**0.64 (0.45, 0.91)**	**0.55 (0.37, 0.81)**	**0.64 (0.42, 0.98)**	0.74 (0.46, 1.18)	0.69 (0.40, 1.19)	0.59
Multivariate-adjusted HR[d] Model 4	1 (ref.)	**0.68 (0.47, 0.98)**	**0.67 (0.45, 1.00)**	**0.57 (0.36, 0.92)**	0.68 (0.39, 1.17)	0.77 (0.41, 1.47)	0.72 (0.32, 1.62)	0.92

SUN, Seguimiento Universidad de Navarra.

[a] Values are HR estimated with Cox regression and 95% confidence intervals (CI).

[b] Model 2: HR adjusted for age (10 categories), sex, body mass index (in 5 categories), total energy intake, following special diets at baseline, physical activity (METs-h/week), alcohol (g/d), smoking (3 categories).

[c] Model 3:HR adjusted for factors in Model 2 plus marital status, body weight changes, years of university education, borderline hypertension at baseline, family history of hypertension, year of entrance to the cohort.

[d] Model 4:HR adjusted for factors in Model 3 plus sodium intake, potassium intake, calcium intake, hours per day spent watching television, analgesic consumption, sugar-sweetened beverages consumption.

Figure S1. Flow chart depicting the selection process among participants of the SUN project to be included in the present analyses.

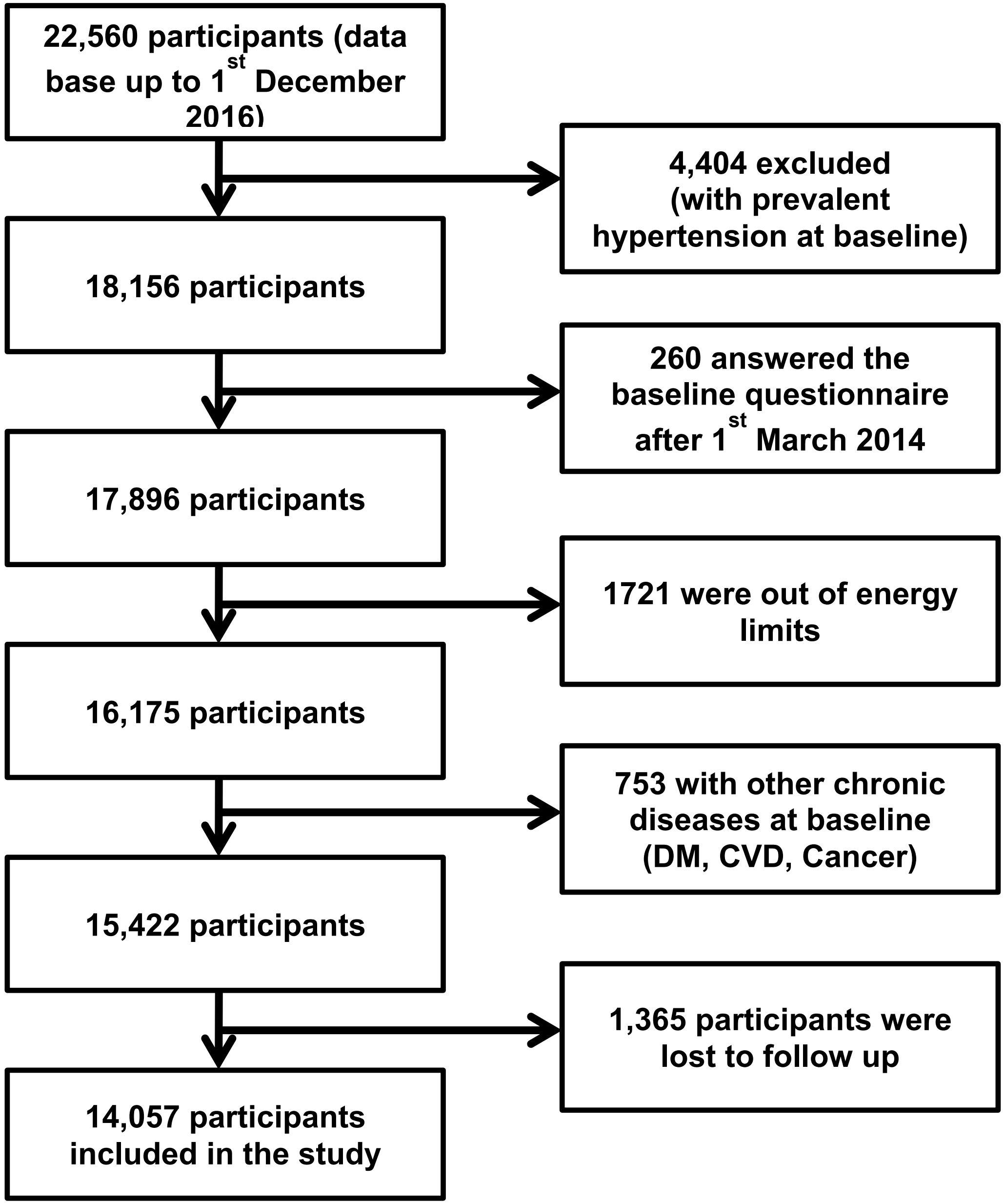

Figure S2. Multivariate-adjusted HR of incident hypertension according to cross-classification by BMI in three categories and magnesium intake in four categories in participants from the SUN project. The model was adjusted for age, sex, BMI, total energy intake, following special diets at baseline, physical activity, alcohol consumption, smoking, marital status, body weight changes, years of university education, borderline hypertension at baseline, family history of hypertension, year of entrance to the cohort, sodium intake, potassium intake, calcium intake, hours per day spent watching television, analgesic consumption, and sugar-sweetened beverages consumption.

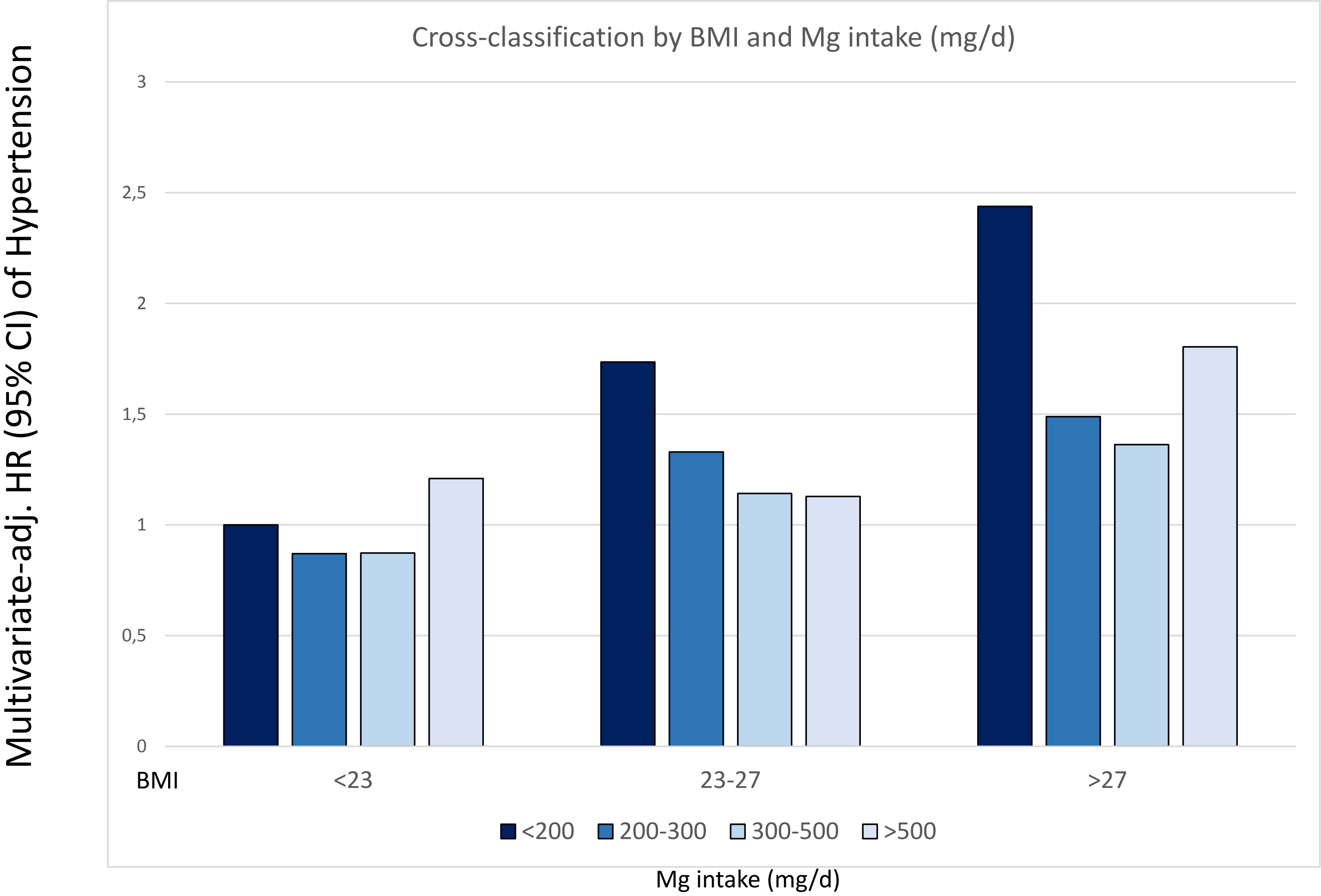

STROBE Statement—Checklist of items that should be included in reports of ***cohort studies***

	Item No	Recommendation
**Title and abstract**	1	(*a*) Indicate the study's design with a commonly used term in the title or the abstract: included in the abstract, lines 24-26
		(*b*) Provide in the abstract an informative and balanced summary of what was done and what was found: lines 23-36
**Introduction**		
Background/rationale	2	Explain the scientific background and rationale for the investigation being reported: lines 40-73
Objectives	3	State specific objectives, including any pre-specified hypotheses: lines 73-76
**Methods**		
Study design	4	Present key elements of study design early in the paper: lines 79-81
Setting	5	Describe the setting, locations, and relevant dates, including periods of recruitment, exposure, follow-up, and data collection: lines 82-92
Participants	6	(*a*) Give the eligibility criteria, and the sources and methods of selection of participants. Describe methods of follow-up: lines 82-92 and Figure 4 (supplementary material)
		(*b*) For matched studies, give matching criteria and number of exposed and unexposed NOT APPLICABLE
Variables	7	Clearly define all outcomes, exposures, predictors, potential confounders, and effect modifiers: lines 102-160 Give diagnostic criteria, if applicable: line 138, reference 53
Data sources/ measurement	8*	For each variable of interest, give sources of data and details of methods of assessment (measurement): lines 102-160 Describe comparability of assessment methods if there is more than one group NOT APPLICABLE
Bias	9	Describe any efforts to address potential sources of bias: lines 83-92 and Figure 4 (supplementary material) lines 102-160
Study size	10	Explain how the study size was arrived at lines 82-92 and Figure 4 (supplementary material)
Quantitative variables	11	Explain how quantitative variables were handled in the analyses. If applicable, describe which groupings were chosen and why: lines 102-160
Statistical methods	12	(*a*) Describe all statistical methods, including those used to control for confounding: lines 161-208

		(*b*) Describe any methods used to examine subgroups and interactions:<br>lines 162-196
		(*c*) Explain how missing data were addressed:<br>lines 89-90 and Figure 4 (supplementary material)<br>lines 135-137
		(*d*) If applicable, explain how loss to follow-up was addressed:<br>lines 89-90 and Figure 4 (supplementary material)
		(*e*) Describe any sensitivity analyses<br>lines 199-205
**Results**		
Participants	13*	(a) Report numbers of individuals at each stage of study—eg numbers potentially eligible, examined for eligibility, confirmed eligible, included in the study, completing follow-up, and analysed:<br>lines 83-92 and Figure 4 (supplementary material)
		(b) Give reasons for non-participation at each stage:<br>lines 83-92 and Figure 4 (supplementary material)
		(c) Consider use of a flow diagram:<br>Figure 4 (supplementary material)
Descriptive data	14*	(a) Give characteristics of study participants (eg demographic, clinical, social) and information on exposures and potential confounders):<br>lines 211-223 and Table 2
		(b) Indicate number of participants with missing data for each variable of interest:<br>lines 82-93 and Figure 4 (supplementary material)
		(c) Summarise follow-up time (e.g., average and total amount):<br>lines 211-212
Outcome data	15*	Report numbers of outcome events or summary measures over time:<br>line 212 and Tables 3 and Table 5 (supplementary material)
Main results	16	(*a*) Give unadjusted estimates and, if applicable, confounder-adjusted estimates and their precision (e.g., 95% confidence interval). Make clear which confounders were adjusted for and why they were included:<br>Table 3, Table 5 (supplementary material), Figure 1
		(*b*) Report category boundaries when continuous variables were categorized:<br>Table 2, Table 3, Table 5 (supplementary material)
		(*c*) If relevant, consider translating estimates of relative risk into absolute risk for a meaningful time period:<br>Table 3, Table 5 (supplementary material)
Other analyses	17	Report other analyses done—eg analyses of subgroups and interactions, and sensitivity analyses:<br>Figures 1, 2, 3 and Table 4<br>lines 225-263
**Discussion**		
Key results	18	Summarise key results with reference to study objectives:<br>lines 324-331

Limitations	19	Discuss limitations of the study, taking into account sources of potential bias or imprecision. Discuss both direction and magnitude of any potential bias:<br>lines 432-455
Interpretation	20	Give a cautious overall interpretation of results considering objectives, limitations, multiplicity of analyses, results from similar studies, and other relevant evidence:<br>lines 332-429
Generalisability	21	Discuss the generalisability (external validity) of the study results:<br>line 436
**Other information**		
Funding	22	Give the source of funding and the role of the funders for the present study and, if applicable, for the original study on which the present article is based:<br>lines 481-484

*Give information separately for exposed and unexposed groups.

Note: An Explanation and Elaboration article discusses each checklist item and gives methodological background and published examples of transparent reporting. The STROBE checklist is best used in conjunction with this article (freely available on the Web sites of PLoS Medicine at http://www.plosmedicine.org/, Annals of Internal Medicine at http://www.annals.org/, and Epidemiology at http://www.epidem.com/). Information on the STROBE Initiative is available at http://www.strobe-statement.org.
